# Helminth Infections, Type-2 Immune Response, and Metabolic Syndrome

**DOI:** 10.1371/journal.ppat.1004140

**Published:** 2014-07-03

**Authors:** Aprilianto E. Wiria, Erliyani Sartono, Taniawati Supali, Maria Yazdanbakhsh

**Affiliations:** 1 Department of Parasitology, Faculty of Medicine, University of Indonesia, Jakarta, Indonesia; 2 Department of Parasitology, Leiden University Medical Center, Leiden, The Netherlands; University of Wisconsin Medical School, United States of America

## The Association between Metabolic Syndrome and Inflammation

Metabolic syndrome (MetS), a disorder of energy utilization and storage, manifests as a cluster of conditions such as hypertension, dyslipidemia, abdominal obesity, and altered glucose metabolism, which increases the risk of type-2 diabetes (T2D) and cardiovascular diseases (CVD). A lifestyle that consists of overeating, high-calorie food intake, and no or irregular physical activity is a risk factor for developing MetS. Therefore, treatment for MetS comprises regular physical activity, diet modification to reduce weight and/or blood glucose, and the use of lipid-lowering drugs.

Recently, evidence is emerging that innate and/or adaptive inflammatory responses might be associated with MetS [1]. Obese people with MetS have higher concentrations of circulating inflammatory markers [2]. Nevertheless, the incidence of CVD is also found to be higher in patients with inflammatory diseases such as asthma [3] or rheumatoid arthritis [4]. It has been shown in vitro that an inflammatory milieu, with pro-inflammatory cytokines and high levels of interleukin (IL) 17 and tumor necrosis factor (TNF), can lead to the development of a prothrombotic state in human endothelial cells [5]. Several studies have shown that controlling inflammation might be linked to improved MetS. A T2D drug, thiazolidinedione (pioglitazone), can improve insulin sensitivity by activation of peroxisome proliferator-activated receptor (PPAR) γ, a type-2 nuclear receptor that regulates fatty-acid storage and glucose metabolism [6]. It was noted that the expression of PPARγ in Foxp3+CD4+ regulatory T cells (Treg) in visceral adipose tissue was essential for the restoration of insulin sensitivity in obese mice after pioglitazone treatment [6]. In addition, subcutaneous injection of adjuvant-free apolipoprotein B100-derived peptides, as atherosclerosis-related antigens, can induce accumulation of atherosclerosis-specific Treg in lymph nodes and prevent development and progression of atherosclerosis in C57BL/6J apoE^−/−^ mice [7]. Along with the finding that statin, a lipid-lowering drug that has beneficial effects on CVD, has an anti-inflammatory role [8], there seems to be a real case for further studies into alternative therapies that interfere with inflammatory processes to combat MetS [9].

## Are Helminth Infections Associated with Metabolic Syndrome?

In high-income countries (HICs), infectious diseases such as helminths (usually associated with bad hygiene or sanitation) are relatively well controlled compared to developing or low-to-middle-income countries (LMICs), especially the rural areas of LMICs. The health problems in the HICs are instead commonly related to MetS. There is increasing evidence that improvements in the infrastructure and control of infectious diseases in LMICs, which are often followed by a decline in infections (including helminth infections), are paralleled by increasing prevalence of various inflammatory diseases such as allergies and autoimmunities as well as of T2D and CVD [10].

Numerous studies have shown an inverse association between helminth infections and inflammatory diseases such as allergies, autoimmunities, and inflammatory bowel disease, but importantly there is emerging evidence that helminths seem to also be associated with a lower incidence of MetS [10]. A study in India has indicated an inverse association between lymphatic filariasis and T2D [11], while a recent report from a rural community in China, which was previously endemic for schistosomiasis, has indicated that past infection with *Schistosoma japonicum* was associated with lower MetS prevalence [12]. The fasting plasma glucose, postprandial blood glucose, HbA1c, and insulin resistance, as well as the level of triglyceride and low-density cholesterol, were inversely associated with self-reported past *S. japonicum* infection. In support of this, we have also found that in Indonesia helminth infections are associated with improved insulin sensitivity (Wiria et al., article, unpublished).

In a study in rural Indonesia, we have shown that intestinal helminth infections in adults were negatively associated with risk factors for CVD, such as body mass index (BMI), waist-to-hip ratio (WHR), and lipid levels [13]. In the same study, we did not find any association between current helminth infections and carotid intima media thickness (cIMT) [13]. This might not be surprising as the cIMT of individuals in this area was very low compared to individuals of the same age living in HICs [14], which would make it difficult for any beneficial effect to be detected. Our finding is supported by another study that investigated atherosclerosis in cadavers [15]. The authors reported that *Opistorchis felineus* infection was associated with lower serum total cholesterol and was a negative predictor of aortic atherosclerosis.

Taken together, it is tempting to hypothesize that helminth infections can protect against MetS, therefore decreasing the risk for subsequent development of T2D and/or CVD.

## What Is the Possible Mechanism behind the Association between Helminths and Metabolic Syndrome?

The ability of helminth parasites to influence the immune system of their host is evident from the association between these infections and immune hyporesponsiveness and the skewing of the immune system toward modified type-2 responses [10]. These characteristic responses are thought to ensure the long-term survival of worms in the human host while benefiting the host by preventing immune pathological reactions [10]. Recent studies have shown that there might be a link between the immune responses that helminths can induce and glucose metabolism. In Table 1, a summary of the expanding body of research on the effect of helminths on various aspects of MetS is presented. Ricardo-Gonzales et al. have elegantly shown that when IL-4 is injected into mice fed a high-fat diet, the signal transducer and activator of transcription 6 (STAT-6) was activated in the liver and attenuated adipose tissue inflammation which in turn lead to improvement of insulin action [16]. Furthermore, Wu and colleagues showed in a landmark study that the nematode *Nippostrongylus brasiliensis* infection in mice could improve insulin sensitivity associated with adipose tissue eosinophilia [17]. In the same report, using noninfected mice, the authors describe eosinophils as the major IL-4 producers in the adipose tissue that sustains alternatively activated macrophages (AAM). These AAMs are able to maintain insulin sensitivity in adipose tissue partly through the production of the anti-inflammatory cytokine IL-10, a mechanism that has been previously demonstrated to counter the effect of TNF on inducing insulin resistance [18].

**Table 1:** Animal models investigating the relationship between helminths and diet-induced metabolic disorders.

Mouse Strain	Helminth Infection/ Extract	Findings	References
(A) ApoE−/− mice or (B) random-bred TO mice	*S. mansoni* infection	• Reduced total serum cholesterol (in both A and B) and HDL and LDL cholesterol (B).	[26]
		• Reduced atherosclerotic lesion development (A).	
(C) ApoE−/− or (D) wild type (WT) C57BL/6 mice	*S. mansoni* eggs that had been frozen	• Reduced total serum cholesterol (C and D) and LDL (C).	[27]
		• No effect on atherosclerotic lesion formation (C).	
		• Enhanced Th2 cytokine responses without affecting Th1 cytokines (C).	
		• Increased percentage of macrophages in peritoneal cavity (C).	
		• Enhanced ability of macrophages to take up LDL but not acetylated LDL (D).	
(E) ApoE−/− mice or (F) TO mice	*S. mansoni* infection, live *S. mansoni* eggs, SEA, and SmECS	• Patent bisexual worm infection reduced serum cholesterol (F) and liver lipids (E and F).	[19]
		• Single-sex worm infection did not significantly reduce serum cholesterol (E, F).	
		• Live eggs, SEA, and SmECS reduced serum cholesterol (F).	
		• Single-sex infection, SEA, and smECS did not affect liver lipids (F).	
WT C57BL/6 mice	*N. brasiliensis* infection	• Improved insulin sensitivity and glucose tolerance.	[17]
		• Decreased perigonadal adipose tissue weight.	
		• Increased periogonadal adipose tissue eosinophils.	
		• Decreased total adipose tissue macrophages.	
(G) WT (H) IL-10−/− or (I) Fxr-α−/− (Nr1h4) C57BL/6J mice	*SEA*	• Improved insulin sensitivity and glucose tolerance in an IL-10-dependent manner (G and H).	[21]
		• Increased circulating IL-4 and IL-10 (G).	
		• Promoted the expression of M2 and metabolic genes and reduced the expression of M1 genes in epididymal adipose tissue (G).	
		• Protected against hepatic steatosis and suppressed lipogenic gene expression (g)	
		• Reduced lipogenesis through activation of Fxr-α (G, I).	
(J) WT (K) STAT6 (−/−) (L) IL-13 (−/−) C57BL/6 mice	*N. brasiliensis* infection	• Reduced diet-induced weight gain and promoted weight loss in obese mice (in J).	[28]
		• Decreased epididymal and brown adipose tissue mass and circulating leptin (J).	
		• Improved glucose tolerance and fasting blood insulin (J).	
		• Decreased intestinal glucose absorption associated with decreased expression of glucose transporters (J).	
		• Reduced hepatic steatosis and affected the expression of genes related to lipid metabolism (J).	
		• Up-regulated gene expression of Th2 cytokines in various organs including epididymal fat (J).	
		• Up-regulated gene expression of the M2 markers *Arg1* and *YM1* in epididymal fat (J)	
		• The weight loss and attenuation of hepatic steatosis were partially or entirely dependent on IL-	
		• 13 and STAT6 (J,K,L)	
(M) LDLR−/− or WT (N) c57BL/6 mice	*SEA*	• Induced up-regulation of splenic Th2 genes (M)	[20]
		• Induced anti-inflammatory peritoneal macrophages but did not affect M2 gene expression (M).	
		• Reduced systemic inflammation at the myeloid level (M).	
		• Reduced plasma cholesterol in the VLDL- and LDL-sized particles (M).	
		• Attenuated atherosclerosis development and decreased plaque necrosis (M).	
		• In vitro, SEA-treated macrophages displayed an anti-inflammatory phenotype and were less adherent to activated endothelium (N).	

Results are shown for a high-fat diet. TO = Tyler's original; WT = wild type; *S. mansoni* = *Schistosoma mansoni*; SEA = *S. mansoni*-soluble egg antigen; SmECS = *S. mansoni* egg culture supernatant; HDL = high-density lipoprotein; LDL = low-density lipoprotein; VLDL = very low-density lipoprotein; Th = T helper; M = macrophage; IL = interleukin; STAT6 = signal transducer and activator of transcription 6; Fxr-α = Fanexoid X receptor alpha; Nr1h4 = nuclear receptor subfamily 1, group H, member 4.

**Figure 1:**
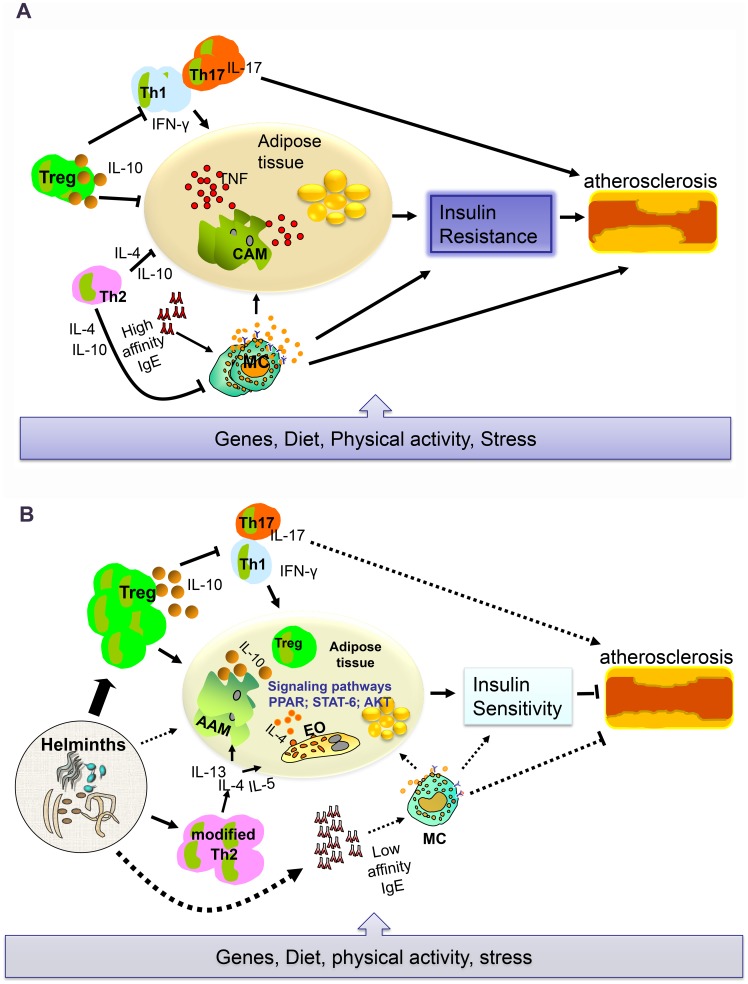
A possible schematic role of inflammatory cells networks on T2D and CVD. (A) The situation in animal models of T2D and CVD without the presence of helminths and in humans living in affluent areas not endemic for helminths. (B) The situation in animal models in the presence of helminths and in humans living in areas endemic for helminths. Although it is known that genes and lifestyle factors are involved in the development of insulin resistance and cardiovascular disease, it is becoming increasingly accepted that the immune system and inflammation play an important role as well. Obese people with metabolic syndrome have a higher degree of inflammation, characterized by increased TNF, a cytokine associated with insulin resistance. When the balance of T cell subsets is disturbed, increased frequencies of pro-inflammatory T cells such as T helper (Th) 1 and Th17 can drive classically activated macrophages (CAMs) which release TNF and, when in metabolic organs such as the adipose tissue, can interfere with insulin signaling. There is also evidence that, under inflammatory conditions, mast cells (MCs) contribute to the pathogenesis of metabolic disorders. High-affinity IgE, present on MCs, can lead to degranulation and initiate inflammation when cross-linked (A). However, the immune system is also endowed with cells such as Th2 and Treg that can exert anti-inflammatory activity and counterbalance the effects of TNF. The balance between pro- and anti-inflammatory activities in the immune system would determine insulin sensitivity. The situation seems to be different in rural areas of LMICs where helminth infections are highly prevalent. Helminths need nutrients from their host for their growth and reproduction, and this might use the energy of their host and therefore forestall obesity and insulin resistance. However, helminths can also lead to the expansion of alternatively activated Th2 and Treg. Th2 cytokines result in increased eosinophilia (EO) and, when in adipose tissue, can lead to the alternative activation of macrophages in this metabolically active organ; the AAMs in turn release anti-inflammatory cytokines such as IL-10. The signaling pathways are currently being dissected, but so far there is evidence that this cascade of events involves the activation of PPAR, STAT6, and/or Akt. Moreover, it has been noted that when the immune system is exposed to chronic helminth infections, EO and MCs no longer behave as pro-inflammatory immune cells, and IgE under these conditions appears to be of low affinity showing poor functional activity in terms of inducing MC degranulation. Thus, in the presence of helminth infections, the immune system is in an anti-inflammatory mode that is considered to be disadvantageous to the development of T2D and CVD (B). The solid lines represent associations based on data available, while dotted lines represent theoretical associations that are yet to be tested.

In a study using apoE−/− mice, it was shown that factors released from *S. mansoni* eggs have lipid-lowering effects [19]. Recently, a report in another model (c57BL/6 LDLR−/− mice) showed that *S. mansoni*-soluble egg antigen (SEA) treatment induces anti-inflammatory macrophages, which may reduce systemic inflammation, attenuates atherosclerosis development, and inhibits plaque necrosis [20]. Moreover, Bhargava et al. [21] have demonstrated that lacto-N-fucopentose III, an immunomodulatory glycan that can be found in human milk and its Lewis-X moiety on parasitic helminths (in SEA), could improve insulin sensitivity by enhancing white-adipose-tissue insulin signaling through induction of IL-10 production. Whether helminth infections lead to similar changes in humans still requires further investigation. In a recent study, we found that total immunoglobulin E (IgE), which is associated with helminth infections, was associated with decreased fasting blood glucose and lipid levels [13]. The scheme that shows the role of inflammation in the development of T2D and CVD in the absence and presence of helminth infections is given in Figure 1.

## Is There More to IgE and CVD?

The association between total IgE and CVD in the absence of helminths has been investigated in animal models. Wang et al. have reported that IgE is associated with CVD by its binding to FcεR1α on macrophages to promote plaque instability [22]. They also reported that IgE levels were higher in subjects with CVD, especially in those with unstable angina and acute coronary events. In an urban area in China, a higher level of IgE and chymase, a mast cell protease, has been reported to be a potential risk factor for T2D [23], [24]. These studies seem to be in contrast to our findings that high total IgE is associated with decreased fasting glucose and lipids [13]. However, recent work has suggested that there are two types of IgE, which provoke different responses when bound to Fcε receptors on mast cells [25]. One is IgE that has a high affinity and can initiate mast cells' degranulation and anaphylaxis when bound to its antigen, whereas another is low-affinity IgE that is functionally less active. Investigating how IgE is involved in the pathogenesis of MetS and how the biological activities of IgE differ in the absence or presence of helminth infections might generate new insights into possible MetS pathogenesis and treatments.

## The Emerging Area of Helminth Infections and MetS

It is tempting to speculate that the possible protective effect against MetS of living in areas where helminths are highly endemic is based on the presence of strong anti-inflammatory and modified responses (Figure 1). To test this hypothesis, sufficiently powered longitudinal studies are needed in the form of a randomized, double-blind, anthelmintic-placebo-controlled trial that can reveal whether deworming leads to MetS. Another method would be to infect patients with MetS with helminths to assess whether the treatment can improve MetS. Only then will it be possible to bridge the gap between the findings in animal models and the situation in humans. Next, it might be interesting to test which helminths or which of their products can be used to modify inflammatory responses as a treatment for T2D or CVD, as is currently being done in the field of allergy, autoimmunity, and inflammatory bowel diseases [10].
